# Role Of Ovarian Metastases In Colorectal Cancer (ROMIC): a Dutch study protocol to evaluate the effect of prophylactic salpingo-oophorectomy in postmenopausal women

**DOI:** 10.1186/s12905-022-02040-1

**Published:** 2022-11-11

**Authors:** R. Van der Meer, I. H. J. T. de Hingh, J. G. Bloemen, L. Janssen, R. M. H. Roumen

**Affiliations:** 1 Department of Surgery, Máxima Medical Center, P.O. Box 7777, 5500 MB Veldhoven, The Netherlands; 2 Department of Surgery, Catharina Cancer Institute, Eindhoven, The Netherlands; 3 GROW - School for Oncology and Development Biology, Maastricht University, Maastricht, The Netherlands

**Keywords:** Colorectal cancer (CRC), Prophylactic salpingo-oophorectomy (PSO), Survival, Number needed to treat, Study protocol

## Abstract

**Background:**

The mean incidence of ovarian metastases (OM) in patients with colorectal cancer (CRC) is 3.4%. The 5-year survival of these patients, even when operated with curative intent, is remarkably low. The lifetime risk of ovarian cancer is approximately 1.3%. Prophylactic salpingo-oophorectomy (PSO, or surgical removal of the ovaries and fallopian tubes) could reduce the number of CRC patients that develop OM after removal of the primary tumor, as well as preventing the occurrence of primary ovarian cancer. Recently, the care pathway for CRC has been changed in several hospitals in line with the updated Dutch guideline. The possibility of PSO is now discussed with postmenopausal CRC patients in these hospitals. The aims of the current study are firstly to estimate the incidence of OM and primary ovarian cancer in postmenopausal patients with CRC, and secondly to evaluate the effect of PSO in these patients.

**Methods:**

An information bulletin and decision guide on this topic was implemented in several Dutch hospitals in 2020. Post-decision outcomes will be collected prospectively. The study population consists of postmenopausal (≥ 60 years of age) patients that are operated with curative intent for CRC. Based on their own preference, patients will be divided into two groups: those who choose to undergo PSO and those who do not. The main study parameters are the reduction in incidence of ovarian malignancies (metastatic or primary) following PSO, and the number needed to treat (NNT) by PSO to prevent one case of ovarian malignancy.

**Discussion:**

This will be the first study to evaluate the effect of PSO in postmenopausal CRC patients that is facilitated by an altered CRC care pathway. The results of this study are expected to provide relevant information on whether PSO adds significant value to postmenopausal patients with CRC.

**Trial registration:**

International Clinical Trials Registry Platform, NL7870. Registered on 2019 July 12. URL of trial registry record: https://trialsearch.who.int/Trial2.aspx?TrialID=NL7870.

Protocol version: 1.0, date 2021 June 8.

**Supplementary Information:**

The online version contains supplementary material available at 10.1186/s12905-022-02040-1.

## Introduction

Intra-abdominal relapse of colorectal cancer (CRC), including ovarian metastases (OM), is a serious event leading to high morbidity and mortality and to a significant loss in quality of life [1, 2]. For CRC patients with OM, including those who are operated with curative intent, the reported median survival is between 12–18 months [1, 3–5] and the 5-year survival rate is about 12–27% [2, 6–10].

### Occurrence of ovarian metastases

The risk of developing OM in patients with CRC has been reported as between 1–8% [1, 3–5, 8, 11–16], with postmortem studies showing a higher incidence of 5–10% [2]. Review of the literature by Pitt et al. [17] revealed the mean risk for development of synchronous and metachronous OM is 3.4%. The risk of developing OM is considerably higher in young or premenopausal patients, with a mean incidence of 5% (range 3 to 50%) [1, 7, 12, 18–21]. 

### Guideline, evidence for prophylactic surgery, and current practice

The Dutch guideline for CRC management was updated in 2019 and includes discussing the role of prophylactic salpingo-oophorectomy (PSO) to reduce the risk of developing OM and primary ovarian cancer in *postmenopausal* patients [22].

To date, only one randomized controlled study (*n* = *155*) has investigated the impact of prophylactic surgery by randomizing patients into one of two groups: prophylactic oophorectomy or non-oophorectomy [11]. This study found no significant difference between the two groups in terms of disease-free survival at 5 years: 78% for the prophylactic oophorectomy group versus 68% for the non-oophorectomy group (*p* = *0.16*). Furthermore, no significant difference in overall survival was found between the two groups (*p* = *0.7*9). However, the statistical power of this study was quite low and hence no firm conclusions could be drawn.

In accordance with the updated Dutch guideline, PSO is now regularly discussed with postmenopausal (≥ 60 years of age) CRC patients in several Dutch hospitals.

### Consequences of PSO

The removal of ovaries in postmenopausal patients can affect the hormone balance. Following oophorectomy, the concentrations of androstenedione and testosterone decrease by 50%, but this does not lead to significant clinical complaints [23, 24]. A recent study showed that postmenopausal status was a risk factor for the development of CRC and adenomas, due mainly to the production of androgens by the ovaries [25]. This hormonal influence may be the reason why CRC is more prevalent in males, with a male-to-female incidence ratio of 4:3 [26].

The removal of ovaries in postmenopausal patients has several potential disadvantages:

- 1) introduction of extra risk during operation, including bleeding or damage to nearby structures such as ureters. However, this risk appears to be low according to a number of mainly gynecological-focused studies [27–29],
- 2) decreased satisfaction with sexual functioning [30].

Proposed benefits of PSO include [31]:

- 1) resection of microscopic ovarian metastases,
- 2) reduced risk of disease recurrence,
- 3) prevention of primary ovarian cancer, which has a lifetime risk of approximately 1.3% in the general population [32].

### Explanation for the choice of comparators and efficacy of PSO

The primary goal of implementing PSO is to improve the health of individual women by preventing the development of ovarian malignancies (primary or metastatic), thus improving disease-free survival, preventing additional treatment-related morbidity, and ultimately improving overall survival. As such, PSO could potentially be a cost-effective procedure, especially from an oncological point of view [26].

Fear of cancer recurrence is an important issue for CRC survivors [33]. A patients’ ability to choose additional prophylactic surgery could be helpful in reducing their fear, since the risk of subsequent metastatic or primary ovarian cancer is removed. Moreover, this supports the practice of “shared decision making”. Beginning in 2020, counseling for PSO (preference of “yes” or “no” to PSO) started to be implemented in the CRC care pathway in several Dutch hospitals. Consequently, the impact of PSO can be prospectively evaluated.

#### Protocol items

The protocol has been written following the Standard Protocol Items: Recommendations for Interventional Trials (SPIRIT) guidance [34]

## Objectives and outcomes (Table 1)

**Table 1:** Primary and secondary outcomes of the study

Outcome	Metric	Time point/period
**Primary**		
Occurrence of ovarian cancer (primary or metastatic)	Incidence	36 months
Number needed to treat to prevent one case of ovarian cancer (primary or metastatic)	NNT	36 months
**Secondary**		
Disease-free survival	DFS	36 months
Number needed to treat with PSO to prevent one case of ovarian cancer (primary or metastatic)	NNT	36 months
Surgery-related morbidity of PSO	Number	36 months
Subsequent intra-abdominal relapse pattern in the non-PSO group	Number	36 months
Abnormal ovaries found during surgery	Number	During surgery
Incidence of ovarian (micro)metastatic disease	Incidence	36 months
Quality of life (EORT QLQ-C30 and EORTC QLQ-CR29)	SUM score	Baseline, 3-, 12-, 24- and 36 months
Repeat surgery for complications (i.e. adhesions)	Numbers	36 months
Long-term overall survival	OS in days	60 months
Preference for PSO	Numbers	36 months
Reversal of decision	Numbers	36 months
Baseline characteristics	Numbers	Before surgery
**Other parameters**		
Type of surgery	Number per type of surgery	During surgery
Operation duration	Minutes	During surgery
Blood loss	Milliliters	During surgery
Pre- and postoperative treatment strategies	Number per type of treatment strategy	Before surgery and 36 months

*NNT* Number needed to treat, *DFS* Disease free survival, *SUM* Single Usability Metric, *OS* Overall survival, *PSO* Prophylactic salpingo-oophorectomy, *EORT QLQ* European Organization for Research and Treatment of Cancer European Union Quality of Life Questionnaire

### Primary objective

The main aim of this study is to determine whether prophylactic bilateral salpingo-oophorectomy conducted in postmenopausal patients aged ≥60 years during surgery for primary CRC reduces the incidence of ovarian malignancies (metastatic or primary) during a three-year follow-up period. Moreover, this study will provide the data necessary to calculate the number needed to treat (NNT) in order to prevent one case of ovarian cancer (metastatic or primary).

### Secondary objectives

• What is the effect of PSO on disease-free survival (DFS) after 3-years of follow-up? What is the concomitant NNT to gain one year of DFS, according to the method of Lubsen *et al.* [35]?
• What is the effect of PSO on surgery-related morbidity?
• In the non-PSO group, what is the incidence and pattern of intra-abdominal relapse, including CRC ovarian metastases and primary ovarian cancer, requiring renewed surgical intervention?
• During primary surgery, how often are abnormal ovaries found that require resection?
• What is the incidence of (micro)metastatic disease in the ovaries of patients with primary CRC?
• What is the effect of PSO on quality of life as assessed using health-related quality of life (HRQL) questionnaires, and effects such as surgery for abdominal adhesions occurring within 3 years?
• What is the effect of PSO on 5-year overall survival?
• What is the percentage of patients who have a preference for PSO (or no PSO) when scheduled for surgery for primary CRC? Within 3 years of their index surgery, how many patients revise their initial decision of no PSO and subsequently undergo PSO?
• Are there differences in the baseline characteristics between patients who choose PSO compared to those who do not? (The baseline patient characteristics include age, ASA-classification, BMI, previous unilateral oophorectomy, comorbidities, and neo-adjuvant therapy (Table 2))

**Table 2:** Baseline characteristics of study patients

Baseline characteristic	PSO	Non-PSO
Age, mean (SD) or median (IQR), years		
ASA classification, No. (%)		
ASA-1		
ASA-2		
ASA-3		
ASA-4		
BMI, mean (SD), median (IQR), kg/m^2^		
Previous (unilateral) oophorectomy		
Yes, unilateral		
Yes, bilateral		
No		
Comorbidities, No. (%)		
Smoking (yes/no)		
Chronic pulmonary disease (yes/no)		
Hypertension (yes/no)		
Diabetes mellitus (yes/no)		
Myocardial infarction (yes/no)		
Transient ischemic attack (yes/no)		
Cerebral vascular accident (yes/no)		
Central arterial disease (yes/no)		
Peripheral arterial disease (yes/no)		
Severe kidney disease (GFR < 30 mg/mmol) (yes/no)		
Neo-adjuvant therapy		
No		
Yes, chemotherapy		
Yes, radiotherapy		
Yes, chemoradiotherapy		

*SD* standard deviation, *IQR* interquartile range, *y* year, *ASA* American society of anesthesiologists, *no* number, *BMI* body mass index, *GFR* glomerular filtration rate

### Other study parameters

Other information will be collected on the type of surgery (colon *vs* rectum, laparoscopic *vs* open), operative duration, intraoperative blood loss, adjuvant treatment strategies, and pTNM classification. Preoperative data are collected during admission to the surgical and/or gynecology department. Data collected during operation is noted in the operative report. Quality of life after the operation is evaluated by questionnaires (part of the standard follow-up / value-based healthcare) given at 3 months and at 1-, 2- and 3-year(s) after surgery. Data collection is performed centrally.

## Methods/design

This prospective, observational cohort study will evaluate short- and long-term effects in post-menopausal patients given the choice to undergo PSO or not during surgery for CRC. As such, two separate cohorts are studied based on the patient’s preference. Cohort 1 includes all patients who had PSO, while cohort 2 includes all patients who did not choose PSO. All patients are followed up prospectively.

### Current practice and study setting

In 2020 an information bulletin and decision guide (Additional file 1) on PSO was implemented in several Dutch hospitals for female patients ≥ 60 years of age. In patients that opted for PSO, prophylactic surgery during CRC will be performed by surgeons, gynecologists, or both (depending on the surgeons’ experience and local hospital policies).

Post-decision outcomes are collected prospectively with standardized variables and data are stored in electronic patient files. These variables will be used for various statistical analyses and will provide evidence as to whether or not PSO adds significant value to postmenopausal CRC patients.

The following website lists all hospitals that contributed patients to this study: https://romic.surgery/ziekenhuizen/.

### Study population and eligibility criteria

**Fig. 1:**
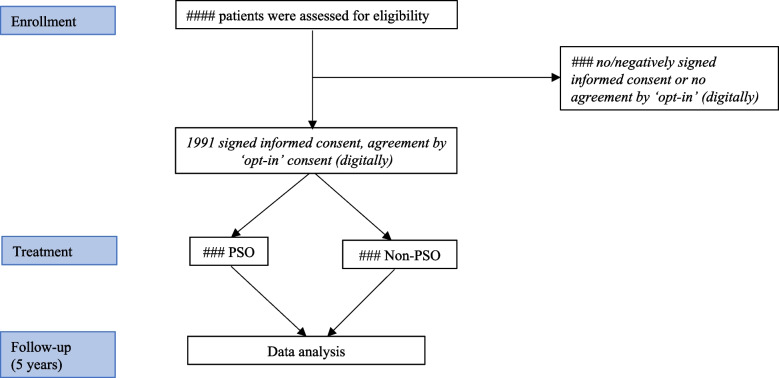
Schematic representation of the cohort. PSO = prophylactic salpingo-oophorectomy

All female patients with CRC who received the information bulletin and decision guide and who signed informed consent (IC) for use of follow-up data are included in this study cohort. Patients are also included when they answered positively to the ‘opt-in’ question for research and education within their electronic health record. Figure 1 shows schematic representation of the study cohort.

#### Inclusion criteria

• Female sex
• Age ≥60 years at the time of CRC diagnosis
• Intended curative resection of colon or rectal cancer, with no evidence of incurable distant metastases
• Informed consent (Additional file 2) or consent by opt-in form (for research and education)

#### Exclusion criteria

• No signed informed consent and no consent by opt-in form (for research and education)
• Surgery with palliative intent
• Known distant metastases preoperatively, or evidence of distant or intraperitoneal metastases during operation, except when curative metastasectomy is considered possible (e.g. for hepatic metastases)

#### Who will obtain informed consent and how

Written, informed consent to participate will be obtained from all participants or, in cases where a positive answer for opt-in (for research and education) exists, data can be used without a written informed consent form. Researchers, registration officers, case managers and/or surgeons will obtain the informed consent.

#### Additional consent provisions for collection and use of participant data and biological specimens

Not applicable.

### Intervention description

This study protocol is not designed for the implementation of a procedure. However, it will be used to evaluate the effect of an existing procedure implemented in the local CRC pathway as follows: PSO *vs*. non-PSO in a female population with CRC. The following items are therefore not applicable: criteria for discontinuing or modifying allocated interventions, strategies to improve adherence to interventions, relevant concomitant care permitted or prohibited during the trial and provisions for post-trial care.

### Randomization, blinding and treatment allocation

Because this study will evaluate the effects of PSO that are facilitated by an altered CRC care pathway, randomization and blinding are not applicable. The two different patient groups are formed based on patient preferences.

### Study procedures

Complications will be scored according to the Clavien-Dindo classification [36, 37]. Furthermore, the Comprehensive Complication Index [38] is a composite score that summarizes the patients’ postoperative wellbeing regarding complications based on the Clavien-Dindo classification. Both scores will be determined after surgery.

Health-related quality of life (HRQL) will be measured using EORTC QLQ-C30 for cancer patients in general, and EORTC QLQ-CR29 specifically for CRC patients. The outcomes will be measured at baseline, at 3 months, and at 1-, 2-, and 3-year(s) after surgery. Differences in outcomes between the two groups will be analyzed statistically.

### Participant timeline

Patients in this study will be enrolled during the period from 07/01/2020 to 07/01/2025. The follow-up period will be 5 years. When a patient withdraws from the study, only the data collected until that time will be used.

### Sample size

The primary study outcome is the occurrence of either CRC metastases in the ovaries or primary ovarian cancer within 3 years after resection of the (primary) colorectal tumor. Based on previous studies, we assume the incidence of synchronous and metachronous colorectal metastases in the ovaries will be 2.0% during the follow-up period [15, 17, 21]. The incidence of primary ovarian cancer is expected to be 0.1% in this period [39]. This gives an overall incidence of 2.1% in cases where PSO is not performed (non-PSO group).

Following PSO, colorectal tumors can no longer metastasize to the ovaries, while the incidence of primary ovarian malignancy should presumably be 0% (PSO group). However, a small risk of primary ovarian malignancy still exists after PSO due to the development of ‘ovarian remnant syndrome’ (ORS) [40–42]. This risk is estimated to be approximately 0.01% for the PSO group.

Based on these assumptions and an alfa of 0.05 with power of 80%, a sample size calculation was performed using an online sample size calculator for comparison of two proportions [43]. This gave a sample size of 371 patients per group, or 742 in total.

Since all eligible patients are not randomized, it is necessary to correct for possible confounders. According to the one-in-ten rule, at least 10 events (of ovarian malignancies) are needed per factor studied in order to achieve sufficient statistical power [44]. Besides PSO, correction will also be made for age as another possible confounder.

Based on current clinical practice, we estimate that about half of all postmenopausal CRC patients undergo PSO during resection of their colorectal tumor. Therefore, we expect the two study groups to be approximately equal in size. The estimated incidence of ovarian malignancies in the total study population will thus be 1.055% (average of 2.1% and 0.01%), thus requiring a sample size of at least 1896 patients (20/0.01055).

Finally, after taking into account a dropout rate of 5%, at least 1996 patients should be recruited into the study.

#### Recruitment

Consecutive CRC patients will be checked for eligibility by their surgeons once they are scheduled for tumor resection surgery. Either the surgeon or the case manager (depending on local logistics) will inform eligible patients about the study at their next visit to the outpatient clinic and provide them with written information. All patients will receive the same written information on the specific issues concerning the study.

Written informed consent for inclusion in follow-up will then be sought from the patient by the involved surgeon or case manager. After informed consent is given, patients are registered with a code (no personal identifiers) in an online case record form using Research Manager [45].

### Data collection and management

#### Plans for assessment and collection of the outcomes

Standardized variables used in the pre-operative records and standardized items during surgery will be used to record the outcomes. An electronic data collection form will be used to capture the information. Participants will be followed up at 3 months and at 1-, 2-, and 3 year(s) after surgery. At each follow-up time, the physician will note whether complications (within ≤ 3 months postoperatively) or recurrent disease have occurred.

#### Plans to promote participant retention and to complete follow-up

There are no additional strategies to promote participant retention since follow-up is the standard of care. In cases where the participant withdraws informed consent, only previously collected data will be used for this study.

#### Data management

Data will be managed by local investigators and local data managers, and local supporting researchers/research assistants. using the online data management system ‘Research Manager’ [45]. Each patient receives a unique study number generated by the data management system. The study number is linked to patient details and is stored in a password-secured file that can only be accessed by the research investigators.

#### Confidentiality

All analyses of study data during the trial period will be carried out in compliance with the relevant regulations for data protection. Personal identifiers will be replaced by a study number generated in ‘Research Manager’. The study data is only accessible by the investigators. Research data that needs to be taken away from the research center will not contain any personal information of the participants. If necessary, government regulatory authorities or ethics committees may access patient data from the study. At the end of the trial, permission from the participants for further storage or for the use of any specimens is already available, since this is included in the signed informed consent form (Additional file 2). Finally, the study results will be published with non-identifiable personal data once the trial has ended.

#### Additional consent provisions for the collection and use of participant data and biological specimens

The collection, processing, and storing of biological specimens will be carried out in accordance with the applicable institutional policies. The use of specimens is described in the patient’s informed consent form (Additional file 2).

### Statistical methods

#### General statistical analysis

Categorical variables will generally be presented as numbers (frequencies) and percentages. Continuous variables will be presented as the mean and standard deviation, or as the median and interquartile range in case of a skewed distribution. The data will only be analyzed and presented quantitatively. Missing data will not be replaced.

#### Statistical analysis of the primary study parameters

For each group, the number of patients who are alive and without evidence of disease relapse after 3 years of follow-up will be determined. Kaplan–Meier curves with the end-point of disease-free survival will be constructed and the log-rank test will be used to compare 3-year disease-free survival rates between groups.

In the case of differences in baseline variables between the two groups, Cox regression analyses including these variables will be performed. Univariate analysis will first be used to identify possible confounders. Multivariate Cox regression analysis will then be performed including ‘group’ (PSO or no PSO), with possible confounders as independent variables and disease-free survival as the dependent variable.

The NNT to prevent one case of ovarian cancer will be calculated according to the method of Lubsen et al*.* [35]. NNT describes the number of patients required to undergo PSO in order to gain 1 year of disease-free survival.

#### Statistical analysis of secondary study parameters

The proportion of abnormal ovaries found during primary surgery that necessitate resection (based on the opinion of the operating surgeon) will be presented as a number and percentage of the total group of study patients. This specific group of patients will be analyzed separately since the need for resection is established before the intervention (PSO) takes place.

Per- and post-operative complications will be presented as numbers and percentages. The number of patients with any complication and the number of patients with a more severely complicated course (Comprehensive Complication Index > 20) will be compared between groups using chi-square tests or Fisher exact tests, as appropriate. In the case of differences in baseline variables between the groups, the number of patients with complications or with a severely complicated course will also be compared using logistic regression analyses that include these variables.

The occurrence of *metastatic spread* to the ovaries (based on pathology reports) will be presented as a number and percentage. Because this can only be assessed in the intervention group, comparison between the groups cannot be made.

The occurrence of relapse of intra-abdominal tumors and the occurrence of primary ovarian cancer are compared between groups using Kaplan Meier analysis and log-rank tests. In the case of differences in baseline variables between the groups, the occurrence of relapse or of primary ovarian cancer will be compared using logistic regression analyses that include these variables.

Generic and disease-specific, health-related quality of life will be measured using the EORTC QLQ-C30 and QLQ-CR29 questionnaires. These will provide continuous variable data that are compared between groups using the student’s t-test or Mann Whitney U-test, as appropriate. Furthermore, linear mixed models for repeated measures will be used to estimate the effect of PSO on the quality of life over time.

#### Other study parameters and methods for additional analyses

All baseline parameters will be compared between groups using either chi-square tests for categorical variables and t-tests, or Mann–Whitney U-tests for continuous variables.

In addition, the surgical substrate (colon *vs* rectum), type of surgery (laparoscopic *vs* open) and use of adjuvant treatment are compared between groups using chi-square tests.

### Oversight and monitoring

#### Composition of the coordinating center and trial steering committee

The data management team consists of local investigators and local data managers.

#### Composition of the data monitoring committee, its role and reporting structure

There will be a research coordinator at each hospital to monitor the trial.

#### Plans for communicating important protocol amendments to relevant parties (e.g. trial participants, ethics committees)

Important protocol modifications will be communicated by e-mail to all relevant parties.

### Ethics and disseminations

#### Dissemination policy

The results of this study will be communicated to all participating hospitals and published in peer-reviewed journals. In addition, the results will be presented at gynecological and surgical conferences.

#### Plans to give access to the full protocol, participant-level data, and statistical code

Not available.

## Discussion

Up until 2019, there was no explicit focus on the role of the ovaries in CRC patients. In our view, however, patients with CRC could gain a benefit from PSO. Apart from the possibility of developing metastases in the ovaries, the risk of developing ovarian cancer at a later stage in life makes PSO a highly relevant issue.

The outcomes of this study will result in continued discussion of the role of PSO. It should also increase awareness among surgeons for the ovaries and salpinges and stimulate them to check the ovaries for possible abnormalities.

In the case of successful completion of this study, evidence should be obtained on different aspects of ovarian malignancies in CRC patients and on the clinical consequences of prophylactic surgery. We will be able to evaluate the impact of recurrent colorectal malignancy, particularly intra-abdominal, as well as the occurrence (or prevention) of ovarian cancer. In addition, we will gain further insights into the disease-free and overall survival of postmenopausal patients with CRC. Based on this new information, we should be able to conclude whether offering PSO to all postmenopausal patients with CRC is beneficial for their oncologic outcome. This conclusion could eventually be incorporated into the CRC guidelines.

Finally, we will gain insight into the long-term effects of both of these operating strategies (PSO or no PSO) on patient quality of life and on complications. Only then will it be possible to balance the considerations that allow informed individual decision-making on this specific issue.

Within the selected hospitals that have altered their CRC care pathway, younger or premenopausal patients are excluded. Therefore, no conclusions can be drawn for this specific group. Since OM appears to be more prevalent in premenopausal patients, research into the effects of PSO on the oncologic outcome of these patients would also be valuable. However, for such a study to be considered, more comprehensive informed consent should be obtained due to the consequences of surgically induced menopause.

At last, the added value of PSO in patients that developed CRC caused by Lynch syndrome, which is the case in approximately 2–4% of all CRC patients [46, 47], remains unanswered by the current study. Although, it is expected that the number needed to treat in this specific population is a lot smaller compared with the general population, because of a lifetime risk of 3–14% for the development of ovarian cancer in patients with Lynch syndrome [48]. A separate substudy regarding this specific population is therefore in preparation.

## Supplementary Information

**Additional file 1.** Patient information bulletin and decision guide regarding colorectal cancer and ovaries.

**Additional file 2.** Informed consent form.

## Data Availability

The datasets and/or analyzed data will be available from the principal investigator (RR) on reasonable request. The Data Management plan and Trial Master File are managed by the principal investigator (RR). Identifying/confidential patient data will not be shared.

## Abbreviations

ASA: American Society of Anesthesiologists
BMI: Body Mass Index
CRC: Colorectal Cancer
DFS: Disease-free survival
EORTC: European Organization for Research and Treatment of Cancer European Union
HRQL: Health-related quality of life
IC: Informed Consent
METC: Medisch ethische toetsing commissie (*dutch*), translation: Medical research ethics committee (MREC)
NNT: Number Needed to Treat
OM: Ovarian Metastases
PSO: Prophylactic (bilateral) salpingo-oophorectomy
pTNM: Pathological Tumor-Node-Metastasis
QLQ: Quality of Life Questionnaire
ROMIC: Role of Ovarian Metastases In Colorectal cancer
WMO: Wet Medisch-wetenschappelijk Onderzoek (*dutch*), translation: Medical Research Involving Human Subjects Act
