# Supplementary material for: Role Of Ovarian Metastases In Colorectal Cancer (ROMIC): a Dutch study protocol to evaluate the effect of prophylactic salpingo-oophorectomy in postmenopausal women
Source: BMC Womens Health. 2022 Nov 11;22:441. doi: 10.1186/s12905-022-02040-1 (PMC9652988; doi:10.1186/s12905-022-02040-1)
Supplement: Supplementary file 1 — Additional file 1. Patient information bulletin and decision guide regarding colorectal cancer and ovaries. [file 12905_2022_2040_MOESM1_ESM.pdf]

Patient information bulletin and decision guide regarding colorectal cancer and ovaries

(version 2021)

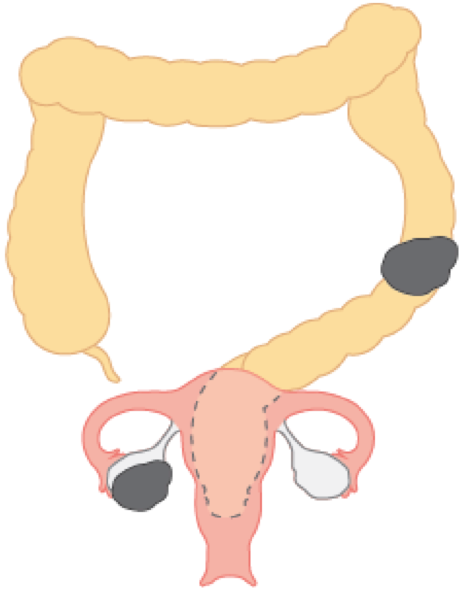

This document was originally written in Dutch

General information

Soon you will be operated for colorectal cancer. During this surgery, the tumor and some surrounding (healthy) tissue will be removed. The surgeon will inspect the abdominal cavity for signs of potential metastatic disease during the procedure. However, metastases are not always visible at the time of surgery. Sometimes there are small metastases present within the ovaries that are not detected by the surgeon or by medical imaging with ultrasound or CT-scan. These metastases can later grow during the follow-up period. One option is to undergo a prophylactic resection of the ovaries and fallopian tubes (adnexa). After this procedure, it is no longer possible for any existing metastases to grow within the ovaries, or for new metastases to develop within the ovaries. It is also possible that the adnexa are not affected by metastatic disease and are therefore removed for no reason. Prophylactic resection of the ovaries does not affect the development of metastases elsewhere in the body.

Why has this information bulletin been developed?

The adnexa are not routinely prophylactically resected in colorectal cancer patients. However, we believe that patients should be informed about the possibility of developing ovarian metastases, so that a decision regarding prophylactic surgery can be made.

In cases where the surgeon suspects (during surgery) that malignant ovarian disease is present, the ovaries are resected as standard procedure. The new situation concerns resection of the adnexa when there is no sign of malignant ovarian disease.

Specifics on the decision guide in this information bulletin

The decision guide helps you to choose the best therapy that is suitable for your situation. The advantages and disadvantages of each therapy are discussed and should prove helpful in making your choice. In this way, you will be well prepared for the next meeting with your physician. Together, you will choose one of the two therapy options.

How can this information bulletin help you?

This information bulletin has been developed for female patients with colorectal cancer who are aged 60 years or more. In these patients, the adnexa can be surgically removed in order to prevent the (further) development of ovarian metastases.

The final decision for prophylactic surgery is made by you. Your medical specialist or nurse practitioner may also be of assistance in making this decision. This information bulletin discusses the advantages and disadvantages of prophylactic surgery.

This information bulletin is not offered to female patients who are younger than 60 years of age.

Background information

Metastases in the ovaries

Colorectal cancer metastases within the ovaries are uncommon. It is estimated that subsequent ovarian metastases occur in only 2 or 3 out of 100 women with colorectal cancer. These metastases mean that another operation must be performed in which the ovaries (and some surrounding tissue) are removed and/or treatment with chemotherapy is necessary.

The consequences of metastases in the ovaries are serious, and only about 1 out of every 5 patients is still alive after 5 years.

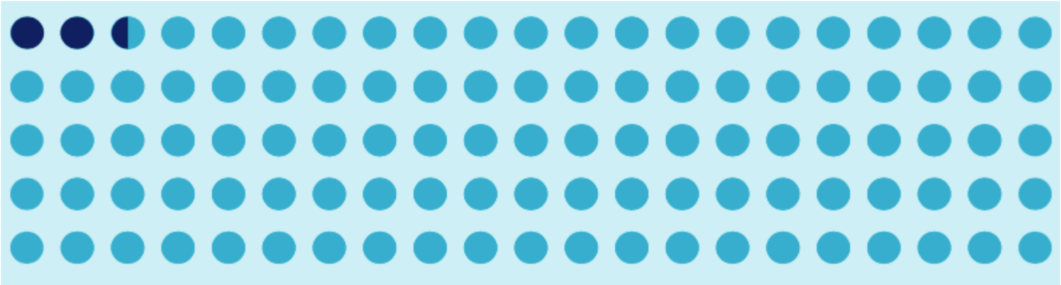

Figure 1: Schematic representation of how much 2-3 in 100 women means

Women before menopause

Removal of the ovaries has more physical consequences for women who are still menstruating (before menopause, also called premenopausal) compared to women who no longer menstruate (after menopause, also called postmenopausal). For this reason and on the advice of the Dutch Society for Obstetrics and Gynecology (NVOG), women under the age of 60 years will not receive this information bulletin. This information is only applicable to women aged 60 years or more.

The treatment and your options

What options do you have?

You will receive the treatment of your choice:

1. Surgery in which only the colorectal tumor is removed
2. Surgery in which the colorectal tumor is removed, as well as the ovaries and fallopian tubes (prophylactically)

The operation

The removal of the ovaries and fallopian tubes takes place during the same operation in which the colorectal tumor is removed. The duration of the operation will therefore be increased by an average of 10-15 minutes. The removal of the ovaries and fallopian tubes is a relatively simple procedure that is performed regularly, and the risk of complications is very small.

Potential disadvantages and complications

Removing the ovaries can have adverse effects. Due to the operation, there is a risk of damage to nearby tissues and organs (such as the ureters) and bleeding can occur. This is estimated to occur in less than 1 in 100 patients, and the possible burden is limited.

In addition, surgical removal of the ovaries can lead to a decreased libido (= sexual arousal).

Option 1:

Surgery in which only the colorectal tumor is removed, with no additional treatment

Important: the ovaries and/or fallopian tubes will be removed if they are visibly abnormal at the time of surgery. This is standard care.

- You will receive the care which is currently offered as standard
- Advantages:
    - The operation duration is not extended
    - There is no risk of complications arising from ovarian and fallopian tube removal
- Cons:
    - There is a risk of developing colorectal cancer metastases to the ovaries later on, estimated at 2-3 in 100 women with colorectal cancer

Option 2:

Surgery in which the colorectal tumor and (prophylactically) the ovaries and fallopian tubes are removed

- The fallopian tubes and ovaries are removed prophylactically to prevent colorectal cancer metastases in the ovaries
- Advantages:
  - Prevents the growth of colorectal cancer metastases in the ovaries
  - No additional surgery is needed later on to remove the ovaries and fallopian tubes, because metastases can no longer occur at these sites
- Cons:
  - Extended operation time (an extra 10-15 minutes on average)
  - There is a risk of complication following the removal of ovaries and fallopian tubes, such as injury to surrounding organs or bleeding. This is estimated to affect less than 1 in 100 women
  - Potentially decreased libido

Compare treatments

	Option 1: No additional treatment	Option 2: Prophylactic removal of the ovaries and fallopian tubes
**Advantages**	1. No extended operation time	1. Prevents the growth of colorectal cancer metastases in the ovaries
	2. No risk of complications from removal of the ovaries and fallopian tubes	2. No additional surgery is needed later to remove the ovaries and fallopian tubes because metastases can no longer occur at these sites
**Cons**	1. Risk of developing colorectal cancer metastases to the ovaries later on, estimated at 2-3 in 100 women with colorectal cancer	1. Extended operation time of 10-15 minutes on average
		2. Risk of complications following removal of ovaries and fallopian tubes (injury to surrounding organs or bleeding, estimated to affect less than 1 in 100 women)
		3. Potentially decreased libido

Important points

- If you do not undergo the additional treatment, you will not have any direct disadvantages from the removal of the ovaries and fallopian tubes, but there is a risk of developing colorectal cancer metastases within the ovaries later on
- Prophylactic removal of the ovaries and fallopian tubes prevents the growth of any colorectal cancer metastases within this organ. Colorectal cancer metastases can still occur elsewhere in the body after prophylactic ovarian resection.

Your preference (what matters to you)

You can discuss the following questions (and answers) with your medical specialist and/or nurse practitioner.

1. I would like to prevent colorectal ovarian metastases

☐ Yes ☐ No ☐ No opinion

2. I am afraid of the risks related to the surgical removal of my ovaries and fallopian tubes

☐ Yes ☐ No ☐ No opinion

3. I value not getting cancer more than the disadvantages of prophylactic removal of my ovaries and fallopian tubes

☐ Yes ☐ No ☐ No opinion

4. I am very worried about the possible loss of libido as a result of ovary removal

☐ Yes ☐ No ☐ No opinion

Your preference

- Which side effects and/or late effects are you most concerned about?
  ____
  ____
  ____

- What is still unclear to you?
  ____
  ____
  ____

Your choice

What will your definitive treatment choice be?

O No additional treatment

O Preventive removal of ovaries and fallopian tubes

How sure are you of your choice?

Very uncertain					Neutral					Very sure
**5**	**4**	**3**	**2**	**1**	**0**	**1**	**2**	**3**	**4**	**5**

Final questions

- Do you know enough about the advantages and disadvantages of the different treatments?

  ☐ Yes ☐ No

- Are you able to determine what is most important to you?

  ☐ Yes ☐ No

- Do you feel that you can make a good and well-balanced decision?

  ☐ Yes ☐ No

- Do you have any additional concerns or questions? Is something still unclear to you?

  ☐ Yes ☐ No

End of information bulletin and decision guide

Authors of this information bulletin

- Máxima Medical Center: Dr. R. Roumen (surgeon), R. van der Meer (PhD candidate), L. Janssen (trial coordinator)
- Other: Surgeons (Prof. Dr. I. De Hingh, Dr. J. Bloemen), gynecologists (Dr. S. Coppus, Dr. P. Geomini), nurse practicioners (D. Lurling, S. v Lankvelt, J. Ophorst), communication and marketing consultant (N. Hermans)

Glossary

- **Adnexa: ovaries and fallopian tubes**
- **Libido: sexual arousal**
- **Postmenopausal: time after menopause**
- **Premenopausal: time before menopause**
- **Prophylactic: preventive**
