# Supplementary material for: Role Of Ovarian Metastases In Colorectal Cancer (ROMIC): a Dutch study protocol to evaluate the effect of prophylactic salpingo-oophorectomy in postmenopausal women
Source: BMC Womens Health. 2022 Nov 11;22:441. doi: 10.1186/s12905-022-02040-1 (PMC9652988; doi:10.1186/s12905-022-02040-1)
Supplement: Supplementary file 2 — Additional file 2. Informed consent form. [file 12905_2022_2040_MOESM2_ESM.docx]

**Additional file 2**

**Informed consent form**

**Evaluation of prophylactic salpingo-oophorectomy in postmenopausal patients with colorectal cancer**

**Background**

Recently the colorectal cancer care pathway has been changed and postmenopausal women can choose for prophylactic surgery (salpingo-oophorectomy) to prevent ovarian malignancies. By this study, we will investigate whether prophylactic salpingo-oophorectomy will be of added value in postmenopausal patients with colorectal cancer. And finally, we will be able to get well-grounded evidence on the real size of this problem and the nature of ovarian malignancies in these patients.

**Data protection and confidentiality**

- I give permission for the gathering of information and the use of my personal data for scientific research
- I know my biological samples will be used and stored for a longer period for scientific research
- I agree that my official cause of death can be requested by the Dutch Central Bureau of Statistics (CBS)
- I know that all collected data will be accessed by all researchers that are involved in this study

**I do agree with all statements that are written below ‘Data protection and confidentiality’**

□ Yes

□ No

Name participant:

Signature: Date: __ / __ / __

----------------------------------------------------------------------------------------------------------------------------------------------------

I hereby declare that I have fully informed the participant about this study.

Name researcher:

Signature: Date: __ / __ / __

----------------------------------------------------------------------------------------------------------------------------------------------------
